# Stigma and psychological distress among pediatric participants in the FD/MAS Alliance Patient Registry

**DOI:** 10.1186/s12887-021-02647-7

**Published:** 2021-04-14

**Authors:** Amanda Konradi

**Affiliations:** Department of Sociology, Loyola University Maryland, 4501 North Charles St., Baltimore, MD 20210 USA

**Keywords:** Fibrous dysplasia, McCune Albright syndrome, Stigma, Self-stigma, Depression, Anxiety, Pediatrics, Children, Neuro-QOL, HADS

## Abstract

**Background:**

Stigma, both enacted and internalized, is part of the illness experience of many chronic conditions / diseases and has been found to increase psychological distress, lower self-esteem, and impact social engagement lowering quality of life (QOL). Stigma among pediatric patients is of particular concern due to its potential impact on identity formation. Using patient data from the online FD/MAS Alliance Patient Registry (FDMASAPR), this study seeks to 1) determine levels of enacted and self-stigma in a pediatric population of fibrous dysplasia (FD) / McCune Albright syndrome (MAS) patients and 2) to explore the relationship between stigma and anxiety and depression.

**Methods:**

This is a cross sectional analysis of deidentified self-report data from 18 pediatric patients. Key analytic variables include the Neuro-QOL stigma short form, the Hospital Anxiety and Depression Scale (HADS), diagnostic category and craniofacial involvement, and select demographics. Sample means and score distributions are examined. Bivariate relationships between stigma, anxiety and depression and patient’s personal and medical characteristics are established through analysis of variance and correlation.

**Results:**

Composite stigma levels for FD/MAS pediatric patients were comparable to those of children with multiple sclerosis, epilepsy, and muscular dystrophy. Self-stigma was more frequently reported than enacted/felt stigma, but few patients indicated complete freedom from either type of stigma. Diagnosis was significantly related to self-stigma. Significant bivariate relationships were found between depression and enacted/felt and self-stigma and between anxiety and self-stigma.

**Conclusions:**

This study establishes the illness experience of pediatric patients with FD / MAS is impacted by stigma and suggests they should be regularly screened for stigma and psychological distress. It supports the integration of clinical psychologists/ therapists in regular patient care, referral of families to advocacy organizations, and indicates that rare disease patient registries can be a useful tool in efforts to improve the QOL of patients.

## Fibrous dysplasia

Fibrous dysplasia (FD) is a rare mosaic bone disease in which fibro-osseous tissue replaces normal bone and marrow producing bones that may bend and fracture or expand beyond their typical limits [1]. Radiographically, the lesions resemble ground glass. Monostotic FD is most common in the rib, skull and femur; polyostotic is most common in the skull, mandible, pelvic bones and femur [2]. Mutations of cells in endocrine tissues also may cause a syndrome, called McCune Albright, that results in café-au-lait marks, precocious puberty; growth hormone excess, hyperthyroidism, hypercortisolism, and renal phosphate wasting [1]. MAS is estimated to comprise 5% of FD patients [3]. Bone lesions can result in a variety of functional problems depending on their location and cause pain [4–6]. New lesions typically stop emerging in adolescence, but the impact of FD/MAS is ongoing over the life course [1].

The difficulty of living with FD/MAS may extend beyond negotiating physical symptoms and emotional sequalae related to them. It may involve living in a body that others note for its differences [7]. The bowing and fracture of bones in the trunk and extremities may affect gait, stance, and ability to move fluidly or impede engagement in activities typical for an individual’s age and gender, sometimes requiring assistive technology. Expansile bone grow may lead to facial asymmetry and distortion of features, impair hearing or vision, and cause the displacement of teeth [6, 8]. Café-au-lait marks may disrupt the continuity of the skin’s appearance. When others note physical differences and impairment resulting from FD/MAS they may stigmatize the whole individual [7, 9] and enact various forms of discriminatory behavior. Even when FD lesions are not visible because they grow inward in the skull, they may affect interaction. For example, those who experience chronic pain and seek strong analgesic medication may experience reproach for drug seeking behavior because they appear “normal” [7, 10].

## Stigma

Stigma refers to the social marking and devaluation of specific qualities of humans, encompassing their physicality, their behaviors, and their membership in social groups [11]. Stigmatization can operate at the level of institutional practice, such that law, policy, and the built world marginalize people with certain traits. Individuals and groups can enact stigma and discriminate against, ostracize, and taunt those with certain devalued traits. And individuals with devalued traits can internalize the negative status and view and/or treat themselves as fundamentally less worthy (self-stigmatize). The different types of stigma tend to be mutually reinforcing [12–17]. Ill individuals often negotiate structural, interactional, and intrapersonal stigma in addition to adapting to their symptoms and treatment regimes.

## Stigma and illness

One sector of illness research is concerned with the nature of stigma experienced by those with appearance affecting conditions, both congenital and emergent/accidental, including cleft lip and palate, cancer, burns, the impact of stigma on life satisfaction and on affected individual’s coping strategies [18–21]. Another sector has focused on stigma experienced by those with more common chronic diseases/conditions, such as, HIV, mental illness, epilepsy, and autism, [22–29]. Rare and orphan diseases that involve a difference of appearance, such as Treacher Collins Syndrome, Crouzon Syndrome, Acromegaly, and vitiligo [30–32] have been studied, but little attention has been focused on how the rarity of diseases/conditions can itself be a unique source of stigma [33].

Studies of a variety of conditions/illnesses have found that enacted or anticipated stigma and self-stigma are associated with negative mental health outcomes, including depression, loss of confidence, low self-esteem, low adherence with medical treatment when compliance might bring on negative reactions from others, and self-isolation that limits an affected person’s use of social support and economic participation [14, 34–37]. Stigma has also been linked to decisions to pursue aesthetic surgical treatment, with limited functional purpose, in order to reduce negative social responses [38].

While there is extensive research on the illness experience of children with craniofacial deformities and an effort to develop a specific quality of life instrument [39–42], more attention has been focused on how stigma affects adults with chronic illnesses than children [43]. Recent studies have found that chronically ill adolescents report feeling different at school and experiencing isolation and exclusion from peer activities [44], especially when their conditions were visible and limiting. Studies also show that chronically ill adolescent’s stigma levels are significantly related to their levels of depression and anxiety [45]. Studies of adolescents with inflammatory bowel disease found illness stigma predicted levels of depression independently and indirectly in relation to their ability to communicate about their illness and their ability to belong to a social group [46, 47]. Attention to the stigmatizing aspects of chronic illness in pediatric patients, especially adolescents, is important because they are developing a sense of self/identity and ways of being in the world that will influence their transition to living as independent adults, attending university and/or acquiring employment [17, 48]. If stigma can be identified early in the illness experience, a variety of resources could be brought to bear to reduce its occurrence and limit its impact on the self of the affected individual and the development of problematic cognitive and psychological sequalae.

The health related quality of life (QOL) of FD/MAS patients is a growing concern of medical researchers [4, 5, 7, 49–52], however the stigma of FD is a new area of empirical research. Adults affected by craniofacial FD (CFD) with and without lesions in other areas of the body have reported experiencing outright discrimination and negative reactions to their appearance, including nonverbal recoil, verbal harassment, isolation, and rejection over the life course [7]. Some have also reported feeling deviant and lesser than others, not just physically different, and engaging in self-isolation [7]. One recent study of adults with CFD found the number of surgeries individual patients had was significantly related to their scores on measures of experienced/enacted stigma [53]. Another found that aesthetics was a commonly reported motivation for craniofacial surgical intervention and those with CFD who did and did not receive surgery had similar long term quality of life scores [54]. Stigma has not been explored in the pediatric FD/MAS population.

This study aims to describe the scope of stigma, both enacted/felt and internalized, in a population of pediatric FD/MAS patients and to explore whether stigma is related to depression, anxiety, and several medical and demographic variables.

## Methods

### Study design

This is a cross sectional analysis of deidentified self-report data from the FD/MAS Alliance Patient Registry (FDMASAPR) [55]. The FDMASAPR is open to individuals from birth to age 70 with fibrous dysplasia and McCune Albright syndrome (fdmasregistry.org) and addresses aspects of the illness experience that are not central to medical treatment but are nevertheless important in the daily lives of patients. It consists of a battery of online questionnaires that document the extent of a participant’s lesions and, if relevant, endocrine involvement, symptoms, surgical and medical treatments received and the motivations for them, quality of life and psychological distress, demographics, and access to treatment. The FD/MAS Alliance recruits participants through its contact database, featuring it in newsletters and in conference updates, and advertising it on the website and through Facebook groups and Twitter. Consenting protocols for the FDMASAPR are reviewed by New England IRB (Needham, MA). All adults (patients and caretakers) complete an electronic consenting process and minors must complete an electronic assent process before entering responses, which creates a digital record of their consent/assent.

In December 2018, when a deidentified FDMASAPR data set was secured, at least one questionnaire had been completed for 83 pediatric patients (1 to 17 years of age). Eighteen, that constitute this sample, had provided comprehensive demographic and bone involvement information and had completed the two key self-report measures required for this analysis. The university IRB declined to subject use of this deidentified data set to further review.

### Measures

The study incorporated the FDMASAPR participant’s age, gender, race, and both parent’s educational attainment levels, reported medical diagnosis, as monostotic FD, polyostotic FD or FD with McCune Albright Syndrome, and presence of craniofacial involvement.

The 8-item Neuro-QOL stigma pediatric self-report short form ascertains patient’s “perceptions of self and publically [sic] enacted negativity, prejudice and discrimination as a result of disease-related manifestations” [56]. The question time frame is “lately” and, all but one question begins with the phrase “because of my illness.” Answers encompass never (1), rarely (2), sometimes (3), often (4) and always (5). The stigma module of the Neuro-QOL was calibrated on children with epilepsy and muscular dystrophy [57]. It was further validated on children with epilepsy; stigma scores discriminated among patients with differing quality of life and severity of illness [51]. The Neuro-QOL stigma short form has not been widely used in pediatric research.

Following the Neuro-QOL scoring protocol, the eight items were combined to produce a composite stigma score (8–40) and normed T-scores were created [56]. For the purposes of calculating the composite, two missing items are replaced with a mean score. Neuro-QOL T-scores above 50 indicate a greater level of stigma than the mean of the clinical reference population of children suffering from neurological conditions [56].

The adult version of the stigma scale has been divided into enacted and self-stigma in FD research [53, 54] as described by Molina and colleagues [58]. Following that practice, the pediatric scale was disaggregated. To create the two sub scales used in this study the questions were first sorted by their apparent meaning. Initially, two groups of questions were created 1, 3, 5, 6 (enacted stigma) and 2, 4, 7 and 8 (self-stigma). Then, a correlation matrix was created to determine if the questions assigned to either scale had significant and high correlations with others that were similarly assigned. As a result of this review, question 8 was determined to reflect anticipation of stigma not self-stigma and the second category was expanded to encompass enacted/felt stigma. Chronbach’s alpha was computed for the two subscales and indicated adequate internal consistency for their use in analysis: enacted/felt stigma (.935) and self-stigma (.816).

The questions that constitute the enacted/felt scale used in this study address other’s demeaning actions or anticipated actions: other’s avoidance (1), teasing (3), unfair treatment (5), and ignoring of the respondent’s good qualities (6) resulting in an expectation of difficult interaction (8). The three questions addressing self-stigma used in this study ask about the respondent’s feelings of difference (7), embarrassment (4) and being left out (2) as a result of their illness.

The Hospital Anxiety and Depression scale (HADS) is a 14 item self-report questionnaire that asks patients how often they have experienced specific feelings in the past week. Answer values range from 0 to 3 and are added to produce anxiety and depression scores, from 0 to 21 [59, 60]. The HADS is validated on general and specific disease populations for adults; it is used extensively in research, especially in Europe [61]. The HADS has been validated on generic populations of adolescents 12 to 17 in the US [62] and Sweden [63]. However, studies of healthy and clinically depressed adolescents and young adults suggest the HADS underpredicts depression in this age range [64, 65]. Two studies have reported significant gender differences in HADS scores among adolescents and young adults [62, 63].

### Analysis

Statistical analysis was conducted with SPSS version 26. Sample means and score distributions were examined and compared to the Neuro-QOL benchmark via t-test. Analysis of variance and Pearson correlation were used to explore bivariate relationships between stigma, anxiety and depression and patient’s personal and medical characteristics.

## Results

### Participant characteristics (see Table 1)

The sample consisted of 18 predominately white (94%) and male (61%) minors with a mean age of 12.9 years (median 13, range 8 to 17). Most of participant’s parents, 83% of mothers and 65% of fathers, had completed college. Polyostotic FD was reported by 67% of participants, followed by McCune Albright Syndrome (22%) and monostotic FD (11%). The majority (61%) did not have craniofacial lesions. All demographic data was complete.

**Table 1:** Demographics and medical characteristics

Age		
Mean	12.9	
SD	2.8	
Median	13	
	Frequency	%
Gender		
Male	11	61
Female	7	39
Race		
White	17	94
Nonwhite	1	6
Mother’s Education		
High school or less	2	11
Some College	1	6
College or more	15	83
Father’s education		
High school or less	0	0
Some College	6	35
College or more	11	65
Diagnosis		
Monostotic	2	11
Polyostotic	12	67
McCune Albright	4	22
Craniofacial		
No	11	61
Yes	7	39

### Stigma

Table 2 depicts univariate analysis of the individual items of the Neuro-QOL Stigma questionnaire and means, standard deviations and medians of the three scales derived from those measures: composite T-score, enacted/felt stigma and self-stigma. Raw total scores ranged from 8 to 24 out of a possible total of 50. Three individuals reported never experiencing any type of stigma (a raw score of 8) and four scored 24, an average of “sometimes” responses. Feeling that their good points were ignored was the least common experience among participants, with 61% of the sample reporting it “never” occurred. “Often” answers were most common in relation to feeling left out. No participants reported they “always” experienced any kind of stigma. The mean composite stigma T-score was 49.56. The median T-score was 52, comparable to consistently responding “rarely.” The minimum enacted/felt stigma raw score was 5 points, reflecting no stigma (6 individuals, 35%); the maximum was 15 points. The mean raw score for enacted/felt stigma was 8.6 points; 34% of the possible total score of 25 points. The minimum self-stigma raw score was 3 points, reflecting no stigma (3 individuals/ 17%) and the maximum score was 12 points. The mean raw score for self-stigma was 7.2 points; 48% of the possible total score of 15 points.

**Table 2:** Stigma univariate statistics

T-score Distributions	37.1	42.8	44.6	46.3	47.7	49.9	50.8	53.3	54	57.7
	3	1	1	1	1	1	1	3	2	4
Score Distributions by Item	Subscale	Never	Rarely	Sometimes	Often	Always	Mean	SD	N	
		(1)	(2)	(3)	(4)	(5)				
1. Others avoided me	felt	8	5	5	0	0	1.88	0.86	18	
2. I felt left out	self	6	2	3	7	0	2.61	1.33	18	
3. Others made fun of me	felt	9	6	3	0	0	1.65	0.79	18	
4. I felt embarrassed	self	7	5	5	1	0	2.00	0.97	18	
5. I was treated unfairly	felt	8	5	2	2	0	1.88	1.05	17	
6. Others ignored my good points	felt	11	5	2	0	0	1.53	0.72	18	
7. I felt different	self	3	4	8	3	0	2.61	0.98	18	
8. I avoided making friends to avoid talking about illness	felt	10	5	3	0	0	1.65	0.79	18	
Stigma Scales	Composite T-score *N* = 18	Enacted/Felt Stigma *N* = 17	Self-Stigma *N* = 18							
Min-Max		5 to 25	3 to 15							
Mean	49.56	8.6	7.2							
SD	7.28	3.8	2.8							
Median	52	7	8							
Minimum score	31.1	5	3							
Maximum score	57.7	15	12							
Mean as % of Possible Total		34%	48%							

Bivariate analysis of stigma scale scores and participant characteristics revealed father’s education and diagnosis were significantly related to stigma T-scores (see Table 3) Bonferroni post hoc tests revealed participants with monostotic FD had, on average, significantly (*p* < .05) higher scores than those with polyostotic FD (+ 13.15) and FD/MAS (+ 16.23). Diagnosis was significantly related to self-stigma as well, although specifics were not established through Bonferroni analysis. No patient characteristics were significantly associated with enacted/felt stigma.

**Table 3:** Stigma x patient characteristics

Variable	Stigma T Score	Enacted/Felt Stigma	Self- Stigma
Age			
*R*^2^	0.032	0.261	−0.035
*P*	0.901	0.312	0.891
Gender			
F	0.002	0.057	0.330
*P*	0.961	0.815	0.573
Mothers Education			
F	1.700	0.484	1.197
*P*	0.216	0.626	0.329
Father’s Education			
F	4.880	3.755	4.087
*P*	0.043*	0.073	0.061
Diagnosis			
F	5.640	1.635	3.718
*P*	0.015*	0.230	0.049*
Craniofacial			
F	0.563	0.759	0.180
*P*	0.464	0.397	0.667

* Indicates significant *p* values

### Anxiety and depression (see Table 4 for univariate and bivariate results)

Anxiety scores ranged from 0 to 14, with a mean of 7.37; ten respondents (55%) scored 8 or more indicating clinical levels of anxiety. Mean depression scores ranged from 0 to 8, with a mean of 2.68; one respondent scored as clinically depressed. Bivariate analysis found participant sex was significantly associated with depression and no patient characteristics were associated with anxiety. Depression scores were significantly and strongly correlated with Stigma T-scores (*R*^2^ = .61) and enacted/felt stigma (*R*^2^ = .75) and moderately correlated with self-stigma (*R*^2^ = .45). Anxiety scores were significantly and strongly correlated with self-stigma (*R*^2^ = .60) and moderately correlated with composite stigma T-scores (*R*^2^ = .50).

**Table 4:** Hospital anxiety and depression scales

	Mean	SD	Median	Range
Univariate				
HADS Anxiety	7.00	3.72	8	14
HADS Depression	2.61	2.50	2	8
Bivariate Relationships				
Variable		Anxiety	Depression	
Age	*R*^2^	0.207	0.288	
	*p*	0.396	0.232	
Gender	F	3.023	6.140	
	*p*	0.100	0.024*	
Mothers Education	F	0.368	0.321	
	*p*	0.698	0.730	
Father’s Education	F	1.014	0.864	
	*p*	0.329	0.366	
Diagnosis	F	0.134	0.106	
	*p*	0.876	0.900	
Craniofacial	F	0.290	1.282	
	*p*	0.597	0.273	
Stigma T-Score	*R*^2^	0.501	0.608	
	*p*	0.034*	0.007*	
Enacted/Felt Stigma	*R*^2^	0.454	0.747	
	*p*	0.067	0.001*	
Self-Stigma	*R*^2^	0.595	0.453	
	*p*	0.009*	0.02*	

*Denotes significant *p* values

## Discussion

Four fifths of the pediatric FD patients in this sample experienced some level of illness related stigma. Discriminatory acts, ridicule and avoidance were reported and anticipated. These children also did not categorically reject the idea that their illness made them different and possibly inferior to others, evidence of stigma internalization. On average their responses to questions associated with self-stigma indicated greater frequency than enacted/felt stigma. Higher levels of self-stigma have been found by other investigators and may indicate that FD, at least in some contexts, has a low level of disruptiveness on interaction and / or is concealable. For example, higher levels of enacted stigma were reported for more socially disruptive muscular nerve disorders than peripheral nerve disorders [36]. The child with FD knows that they are subject to being discredited and rejected even when it does not occur. Children who fear being publicly exposed as lesser may also engage in defensive isolation, which minimizes the likelihood of direct discrimination. The finding of an association between diagnosis and the composite stigma measure and self-stigma, but not enacted/felt stigma is consistent with this. Children may associate greater physical involvement with FD with greater deviation from “normal”; although their physical variability may not be readily apparent to others. The bivariate relationship between stigma and father’s education may reflect the impact of social class position on likelihood of facing discrimination, such that children with less educated fathers have lower social status and generally face more discrimination. Or, less educated fathers may be less accepting of medically complex children, especially boys who cannot live up to physical ideals of masculinity and thus serve as a conduit of stigma (parent reader communication).

This study found the mean composite stigma score for children with FD was similar to children with serious chronic neurological conditions. Higher scores for enacted/felt stigma were associated with higher levels of depression, but not with anxiety. Self-stigma was associated with anxiety and depression. While this cross-sectional study does not establish a causal relationship, the literature from chronic illness suggests that stigma increases psychological distress [15, 45, 46]. These results point to the desirability of incorporating screening for stigma into the structure of pediatric care for FD [66]. Adults with FD/MAS reported a range of responses to enacted and felt stigma, including self-isolation [7]. There is reason to be concerned about adolescents turning to strategies of self-isolation as a protective measure, as it can affect their ability to form relationships and may impact their later participation in higher education and employment [67].

Patients and families should be aggressively referred to support groups and patient organizations [17, 68]. Such communities can offer proactive coping strategies and provide a disability rights orientation that may help encourage frank discussion instead of denial and silence to normalize the patient identity and possibly limit internalization of stigma, as well as support parents of affected children [17, 69–71]. Programs can help children and adolescents with FD/MAS manage their illness identity and develop skills for troubled interactions with others [7]. Parents can also be helped to manage courtesy stigma and the stigma of being a bad parent, which can influence their parenting style in ways that may be detrimental to the identity development and coping strategies adopted by their children [72–75]. Given the rarity of FD/ MAS and the geographic dispersal of patients, online strategies for service delivery should be explored, such as the CBT program FaceIT [76]. Finally, attention should be given to ensuring that medical providers, who have an immense power to shape how children/adolescents view themselves and their condition, consistently use language that is not demeaning and that emphasizes abilities rather than deficits (parent reader communication).

Evidence supports psychosocial intervention. Research has established the efficacy of a combination of psycho-social education and cognitive behavior therapy (CBT) [66, 77–79] or narrative practices [80] to reduce self-stigmatization among those with chronic stigmatizing illnesses. Studies show that providing social skills training improves the ability of children with craniofacial differences to initiate interactions [81]. Social skills training, CBT to heighten self-esteem, and guidance to manage emotional reactions, have also been found effective to reduce teasing [82, 83] of/by children.

This study has several limitations. This pediatric sample from the FDFPR is small with low power, perhaps too small to capture the significant impact of demographics and disease characteristics on stigma, anxiety and depression [72]. The sample is also skewed to white children with well-educated parents limiting its generalizability. Pain was not included as a control variable although chronic pain has been found to correlate with stigma [84] and with depression [85, 86]. Finally, the cross-sectional design precludes establishing causality.

This study may also underestimate the level of psychological distress associated with FD/MAS for several reasons. The Neuro-QOL stigma measure is limited; it does not encompass the full scope of enacted and self-stigma. For example, it does not address appearance related stigma, which can be an important issue for those with craniofacial FD or with visible café-au lait marks. The HADS attends only to aspects of depression related to the loss of pleasure response (anhedonia) [59] and does not record aspects of feeling sad or blue often associated with medical illness [87]. It has also been reported to under estimate depression in adolescents [64, 65]. Like prior research, this study found participant gender significantly contributed to explaining depression [62, 63]. It is possible that the greater proportion of males in this sample skews the reported mean levels of depression downward.

This study examines the existence of stigma and its relationship to anxiety and depression through short quantitative screening measures. Further investigation of stigma among pediatric FD patients using more comprehensive measures should be undertaken. Qualitative research is also needed to develop an understanding of the coping strategies that FD / MAS patients have developed to deal with enacted/felt and self-stigma and the parenting styles and educational accommodations that provide the most protection to children [73].

## Conclusion

This first study of stigma in a population of pediatric FD / MAS patients found that they experienced it at levels comparable to children with other chronic and stigmatizing diseases/conditions. Stigma among pediatric patients with FD / MAS was also significantly associated with measures of anxiety and depression. These results speak to the potential of voluntary rare patient registries to address the broader illness experience and improve quality of life [88–90]. Registries created and sustained by advocacy organizations, including patients and their caretakers, can provide leadership by incorporating psychological and social measures that can lead to more holistic treatment over the life course.

## Data Availability

The data that support the findings of this study are available from the FD/MAS Alliance but restrictions apply to the availability of these data, which were used under license for the current study, and so are not publicly available. Data are however available from the author upon reasonable request and with permission of the FD/MAS Alliance.

## Abbreviations

CFD: Craniofacial fibrous dysplasia
FD: Fibrous dysplasia
FD/MASA: FD/MAS Alliance
FDMASAPR: FD/MAS Alliance Patient Registry
HADS: Hospital Anxiety and Depression scale
MAS: McCune Albright syndrome
